# Patient Safety in the Surgical Field: A Cross-Sectional Study Among Al-Baha University Medical Students

**DOI:** 10.7759/cureus.47923

**Published:** 2023-10-29

**Authors:** Khalid A Alzahrani, Yasser Kofiah, Wafaa S Taishan, Sarah S Taishan, Hind A Alghamdi, Ramy Samargandi

**Affiliations:** 1 Department of Surgery, Faculty of Medicine, Al-Baha University, Al-Baha, SAU; 2 Faculty of Medicine, Al-Baha University, Al-Baha, SAU; 3 Department of Orthopedic Surgery, Centre Hospitalier Régional Universitaire (CHRU) de Tours, Tours, FRA; 4 Department of Orthopedic Surgery, Faculty of Medicine, University of Jeddah, Jeddah, SAU

**Keywords:** surgery, medical students, surgical complications, malpractice, patients safety

## Abstract

Background

Patient safety and quality of surgical care are crucial in healthcare. Adequate knowledge and attitudes among healthcare providers regarding differentiating malpractice from surgical complications are essential for preventing adverse events. We aimed to assess medical students' knowledge and attitudes toward patient safety in surgical procedures.

Methodology

A cross-sectional study was conducted among clinical years of medical students and interns at Al-Baha University, Saudi Arabia, from June 15, 2023, to August 1, 2023. Exclusion criteria were students from basic years, pharmacology students, applied medical science, dental students, and students from other universities. A self-administered questionnaire collected data on participants' demographics, knowledge, attitudes, and practices related to patient safety in surgical procedures.

Results

A total of 271 medical students participated, exceeding the target sample size of 181. Participants' ages ranged from 20 to 28 years, with the majority between 23 and 25 (60.5%). Males accounted for 63.8% of participants. The largest group was fourth-year students (31.7%), followed by interns (23.6%), those in sixth year (23.2%), and those in fifth year (21.4%). Moreover, 82.7% of participants demonstrated good knowledge of patient safety concepts. The highest level of knowledge was noted among fourth-year students (89.5%), and the lowest was among fifth-year medical students (75.9%), but was not statistically significant (p=0.701). Most participants demonstrated appropriate attitudes and practices (83.6%); however, 9.2% refused to perform surgery on a patient with active hepatitis B due to concerns for their own safety.

Conclusion

The majority of participants exhibited suitable knowledge and attitudes toward patient safety in surgical scenarios, but there was deficient knowledge among fifth- and sixth-year students. Moreover, a negative attitude regarding patient safety was noticed, exemplified by refusing surgeries on patients with active hepatitis B.

## Introduction

The World Health Organization (WHO) defines patient safety as “the prevention of errors and adverse effects to patients associated with health care” and “to do no harm to patients” [1,2]. In addition, medical malpractice is defined as professional negligence on the part of a healthcare provider when the care they provide for a patient is subpar compared to the accepted standard of care in the medical community and results in harm or death for the patient, with medical error accounting for the majority of cases [3]. There are millions of patients globally who suffer disabilities, injuries, or death each year due to unsafe medical practices [4]. This has led to the wider recognition of the importance of patient safety, the incorporation of patient safety approaches into the strategic plans of healthcare organizations, and a growing body of research in this field [5].

Healthcare must prioritize patient safety [6], which is strongly being incorporated into medical school curricula, and it is necessary to assess the efficacy of various patient safety education delivery methods [7]. Therefore, patient safety, according to WHO, attempts to prevent and reduce risks, mistakes, and harm that patients experience during the provision of healthcare [8,9]. Medical students are crucial for the development of the healthcare system as a whole, although they are frequently undervalued as key contributors to ensuring this safety [10]. To do this, healthcare staff need to have positive attitudes toward this important topic [11]. Surgical malpractice is a sort of negligence that includes injuries brought on by "neglect or unskillful management" on the part of a physician, in breach of the confidence that was placed in that professional [12]. On the other hand, in each surgical department, complications can occur [13]. Postoperative complications are unintended side effects of surgery, and it is considered a major source of worry since they have a negative impact on the standard of surgical care and the safety of the patient [14]. These range from seemingly trivial episodes that end without any harm to more significant ones that may endanger life, require repeated treatments, lengthen hospital stays and expenses, and occasionally result in death or disability [15]. In addition to the physical injury they inflict, surgical complications can affect patients' quality of life by causing psychological stress [16]. It is crucial to know the difference between common surgical complications and malpractice.

The aim of the current study is to assess the gaps in knowledge about the level of awareness of medical students regarding surgical patient safety, including common surgical complications, malpractice, and attitudes toward malpractice among medical colleges of Al-Baha University, Al-Baha, Saudi Arabia.

## Materials and methods

This was a cross-sectional study conducted from June 15, 2023, to August 1, 2023, to assess the awareness of medical students and interns of Al-Baha University regarding surgical patient safety along with the difference between common surgical complications, malpractice, and attitude toward malpractice.

Inclusion criteria

Female and male medical students in their clinical years and interns at Al-Baha University agreed to participate in the current survey study.

Exclusion criteria

The study excluded participants who fall under the following criteria: students in their basic years of medical education, pharmacology students, individuals pursuing applied medical sciences, and dentistry students. Additionally, students from other universities were not considered for inclusion in the study.

Sample size calculation

The total number of clinical years of medical students and interns was approximately 340. Using Cochran’s equation together with population correction, the required sample size was estimated to be 181.

Data collection and analysis

An anonymous, self-administered validated electronic questionnaire was distributed through social media among the clinical year’s medical students and interns of Al-Baha University. The questionnaire was available in two languages, English and Arabic, and the participants were free to choose the language they preferred. The questionnaire collected demographic data, knowledge about the difference between common surgical complications, malpractice, and attitudes toward malpractice. The questionnaire also included questions about patient safety.

Data were coded and entered into the Statistical Package for Social Science (SPSS) computer program for analysis (v.26; IBM Corp., Armonk, NY). The questionnaire was prepared depending on the previous literature review and consisted of three parts. The first part assessed the demographic factors of the participants (four questions considered age, gender, marital status, and academic level), while the second part consisted of 13 questions, including two questions about the definition of malpractice and surgical complications and nine questions about cases that participants had to identify as malpractice or surgical complications. Part three consisted of seven situations of malpractice to determine the attitudes and practices of participants. For all questions, frequency and percentage were used for descriptions of the results. For the knowledge section, for each correct answer, the participant was rewarded with one point to form a score between 0 and 17, where having a score of 12 or above is considered adequate. The chi-square test was used for assessment of the association between knowledge and demographic factors. All statements with a p value of lower than 0.05 were considered significant.

Ethical considerations

The study was conducted after obtaining ethical approval from the Institutional Research Board of Al-Baha University number (REC/SUR/BU-FM/2023/33R). The participants were informed about the study aims and assured of data confidentiality, and consent was obtained from each participant before participating in the study.

## Results

In the current study, we were able to collect data from 271 medical students who participated in the study. The age of the participants ranged between 20 and 28 years old where the majority of participants fell in the age range of 23-25 (60.5%, *n*=161). In addition, 63.8% (*n*=173) of the participants were males (with male to female ratio of 1.76:1), and 94.8% (*n*=257) of the participants were single. In terms of academic year, the largest group was in their fourth year (31.7% of participants, *n*=86) (Table 1).

**Table 1:** Demographic factors of the participants

		n	%
Gender	Male	173	63.8%
	Female	98	36.2%
Age	20-22	74	27.8%
	23-25	161	60.5%
	26-28	31	11.7%
Marital status	Single	257	94.8%
	Married	14	5.2%
Academic year	Fourth year	86	31.7%
	Fifth year‎	58	21.4%
	Sixth year	63	23.2%
	Intern	64	23.7%

**Figure 1:**
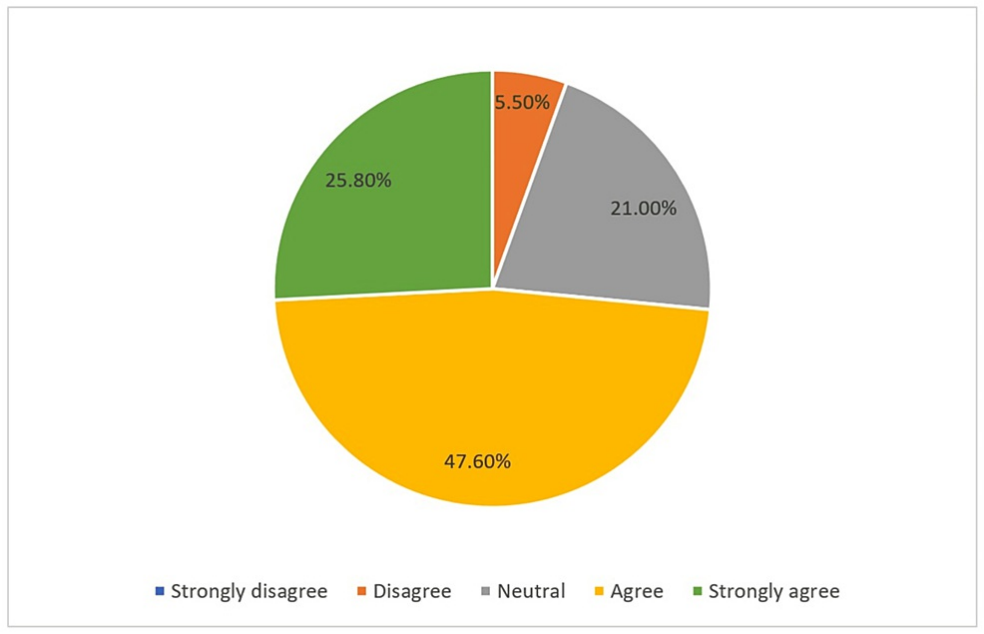
Scale of participants' knowledge regarding differentiating malpractice from surgical complications

Additionally, 25.8% of the participants (*n*=70) strongly agreed that they had excellent knowledge regarding differentiating malpractice from surgical complications, while 47.6% (*n*=129) had lower confidence in their knowledge (Figure 1).

However, 82.7% of participants had adequate knowledge about the difference between malpractice and surgical complications, where the participant was rewarded with one point to form a score between 0 and 17, where having a score of 12 or above is considered adequate. In addition, it was found that participants were able to differentiate between postoperative complications and medical malpractice in most of the cases with correct answers ranging between 57.6% (*n=*156*) *and 92.6% (*n=*251*)*. In one case scenario, 42.4% of the participants (*n=*115*) *would not be able to detect the postoperative complications of hoarseness of voice due to left recurrent laryngeal nerve injury after thyroidectomy (Table 2). No demographic factors of the participants were found to have a significant impact on the knowledge of the participants. However, it was noticed that females, younger participants (20-22 years old), married, and those in fourth year and interns had slightly higher levels of knowledge than other groups but without statistical significance (Table 3).

**Table 2:** The relation between knowledge and demographic factors

		Inadequate		Adequate		P-value
		n	%	n	%	
Gender	Male	35	20.2%	138	79.8%	0.095
	Female	12	12.2%	86	87.8%	
Age	20-22	7	9.5%	67	90.5%	0.090
	23-25	34	21.1%	127	78.9%	
	26-28	6	19.4%	25	80.6%	
Marital status	Single	46	17.9%	211	82.1%	0.301
	Married	1	7.1%	13	92.9%	
Academic year	Fourth year	9	10.5%	77	89.5%	0.701
	Fifth year‎	14	24.1%	44	75.9%	
	Sixth year	15	23.8%	48	76.2%	
	Intern	9	14.1%	55	85.9%	

**Table 3:** The response of the participants toward different situations

	Postoperative complications		Medical malpractice	
	n	%	n	%
A cholecystectomy was done for a 56-year-old patient. 10 days after, he developed a fever and abdominal pain. On CT scan there was an intra-abdominal abscess	238	87.8%	33	12.2%
A 75-year-old man was suffering from end-stage renal cell carcinoma in his right kidney. the left nephrectomy was done instead of the right nephrectomy	20	7.4%	251	92.6%
A thyroidectomy was done by a specialized thyroid surgeon for a 35-year-old woman. After the surgery, the patient presented with hoarseness of voice due to left recurrent laryngeal nerve injury:	156	57.6%	115	42.4%
A pelvic mass removal was done for a female patient, a day after, she started to complain of abdominal pain and vaginal bleeding, the surgeon assures her that there is nothing to worry about, and hours later she died due to internal bleeding:	39	14.4%	232	85.6%
Surgical fixation was done on a 40-year-old female patient for a knee fracture. A week later, she developed leg pain with swelling, which was diagnosed as deep venous thrombosis	244	90.0%	27	10.0%
Cesarean section was done for a female patient, 10 days after, she started to complain of abdominal pain and fever, and a foreign body was detected by CT scan, during the exploratory laparotomy the gauze was found and removed	26	9.6%	245	90.4%
A 16-year male patient was admitted for tonsillectomy during patient transportation to the operating room; they relied on the patient's first name and bed number instead of his full name and Identification number and the surgery was performed for the wrong patient	23	8.5%	248	91.5%
A cesarean section was done for a female patient, and a couple of days after, an investigation revealed a subcutaneous hematoma that was managed conservatively 5 days later when the patient presented with redness and purulent discharge at the surgical site	224	82.7%	47	17.3%
A heart valve replacement was done for a 65-year-old patient. Three days after, he began to complain of chest pain with a productive cough, and high fever. that diagnosed with postoperative pneumonia.	247	91.1%	24	8.9%

**Figure 2:**
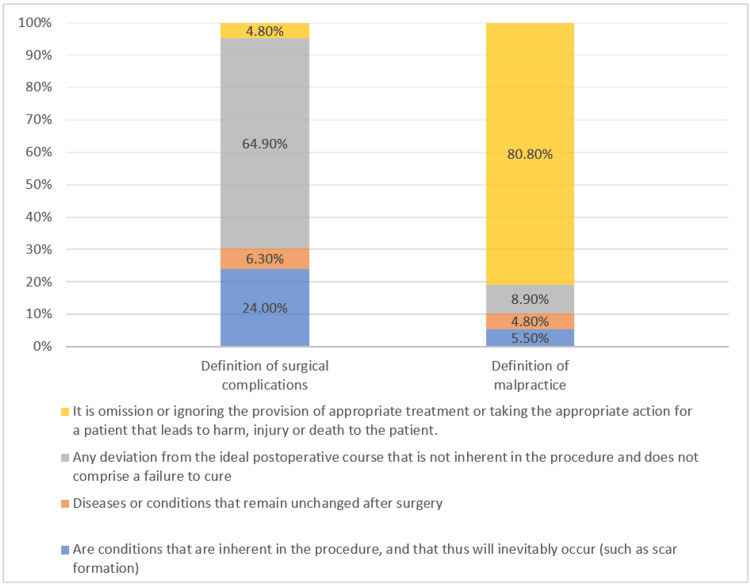
Definition of both malpractice and surgical complications

For surgical complications, 64.9% of the participants (*n*=176) correctly defined surgical complications as "Any deviation from the ideal postoperative course that is not inherent in the procedure and does not comprise a failure to cure." For malpractice, 80.8% of participants (*n*=219) correctly chose the definition "It is omission or ignoring the provision of appropriate treatment or taking the appropriate action for a patient that leads to harm, injury or death to the patient" (Figure 2).

Considering the attitude of the participants toward patients’ safety, 83.6% demonstrate a proper attitude toward patient safety. Interestingly, 9.2% of participants (*n*=25) would refuse to perform the surgery to protect themselves and other patients regarding patients with active hepatitis B (Table 4).

**Table 4:** The attitude and practice of the participants toward different situations

		n	%
Prior to the left hepatic lobectomy, the resident doctor noticed that the surgeon was unwell, and the surgeon told him that he was diagnosed with tuberculosis, and he asked him to doesn't tell anyone.	Report it to the health authorities	221	81.6%
	Ask for a sick leave to protect himself	12	4.4%
	Ask another resident and discuss the situation	33	12.2%
	Non-disclosure, and complete the process procedures	5	1.8%
An old patient with a complicated past medical history was admitted for hemicolectomy; prior to the surgery, a cardiovascular system assessment was done by the anesthesiologist, and the surgeon was advised to reschedule the surgery. The surgeon refused and showed a bad attitude.	Listen to anesthesiologist, re-asses the patient, and reschedule the surgery	243	89.7%
	Ignoring the anesthesiologist and do the surgery to avoid worsening of the condition	9	3.3%
	Apologizing to anesthesiologist and trying to convince him to perform the surgery	7	2.6%
	Call another anesthesiologist to assist in the surgery	12	4.4%
A 25-year-old male patient with an active hepatitis B is scheduled for an appendectomy. What’s the appropriate behavior should be done by the surgeon?	It is better to perform the surgery as the last patient of the day in order to prevent the infection spread	218	80.4%
	Transferring the patient to another hospital to protect the other patients	17	6.3%
	refuse to perform the surgery to protect himself and other patients	25	9.2%
	Perform the surgery as the first patient of the day	11	4.1%
A surgical repair was done for a 10-month-old male with a right side inguinal hernia by an unspecialized surgeon. During the surgery, the right vas deferens and the testicular blood vessels got injured. What is the appropriate behavior that should be done by the surgeon?	Tell the patient's family and report that to health authorities.	229	84.5%
	Tell a colleague and discuss the situation.	36	13.3%
	Tell the family that was a regular surgical complication.	4	1.5%
	No need for reporting.	2	0.7%
During an operation or an invasive procedure, the medication or solution in a syringe, medicine cup, or bowl does not need to be labelled if it is the only medication in the field.	Strongly disagree	183	67.5%
	Disagree	45	16.6%
	Neutral	17	6.3%
	Agree	12	4.4%
	Strongly agree	14	5.2%
Before any surgical procedure, the patient's bed or room number can be used as a reliable source for patient identification.	Strongly disagree	183	67.5%
	Disagree	38	14.1%
	Neutral	20	7.4%
	Agree	15	5.5%
	Strongly agree	15	5.5%

## Discussion

Surgical malpractice can lead to devastating consequences, whether for patients or the entire healthcare system [17]. Misdiagnosing a surgical complication as malpractice has a negative impact on the quality of care because the stress on the surgeon will increase. Consequently, the patient's outcome will be affected. For instance, it might result in unnecessary legal action against a healthcare professional [16]. On the other hand, neglecting to recognize malpractice can result in a lack of accountability and a failure to address systemic issues that may be placing patients at risk [18-20]. Therefore, awareness about this issue should be integrated into the early stages of medical education [11]. In our study, we found that the majority of participants (82.7%) had adequate knowledge of the distinction between malpractice and surgical complications as well as patient safety in the surgical field. This conclusion is positive because it indicates that the medical education system provides students with a solid foundation for comprehending the differences between these two concepts. Another study conducted in Saudi Arabia, which focused on knowledge and attitudes toward patient safety in general among a group of undergraduate medical students, showed that more than half of the participants (52.7%) self-rated their general knowledge of patient safety as good, compared to 27.3% for the specific knowledge issues score [21]. On the other hand, Kamran et al. reached a similar conclusion to our study, where medical students had a positive attitude toward patient safety; however, there were misconceptions about the causes of medical errors and error disclosure among students [22]. Furthermore, one study was conducted in Ethiopia, but it focused on surgical residents; the conclusion drawn from that study was that the overall awareness regarding medical malpractice was relatively low [23].

Our study found that the association between each demographic factor and adequate knowledge levels is not statistically significant. This suggests that demographic factors, such as gender, age, marital status, and academic year, do not have a significant impact on knowledge levels. Although there is a relationship between knowledge level and demographic factors, but it is not strong enough to be statistically significant. However, fourth-year students (89.5%) have the highest percentage of adequate knowledge, while fifth-year (75.9%) and sixth-year (76.2%) students have the lowest percentage of adequate knowledge. One possible reason for this difference is the integrated system applied by the College of Medicine at Al-Baha University, where patient safety was illustrated to the students in the fourth year, and there was minimal revision later on. This finding is consistent with a previous study that examined the knowledge of patient safety among surgical trainees [23]. A similar trend observed in that study indicated that younger surgeons had a higher level of knowledge about patient safety compared to their older counterparts [23]. This trend can be attributed to the more recent exposure to patient safety education and training among younger individuals.

The lack of a significant correlation between knowledge and academic standing, specialization, years of experience, or degree of training suggests that these factors may not be the primary drivers of differences in knowledge levels. Instead, the academic year appears to be a more influential factor in this context. Based on these findings, it is important for medical educators to consider the academic year-related differences in knowledge and tailor educational approaches accordingly. Students in advanced academic years may require additional support or alternative teaching strategies to enhance their understanding of patient safety concepts. This could involve the reevaluation or reactivation of the patient safety education curriculum to ensure its effectiveness across different academic year groups.

This study's findings reveal that the majority of participants have suitable attitudes and practices toward patient safety. However, there were some concerning outcomes, such as the fact that 9.2% of participants would refuse to perform surgery on an active hepatitis B patient in order to protect themselves and other patients. It is crucial to emphasize that patients with hepatitis B have the right to receive appropriate medical treatment and surgical interventions, just like any other patient. Refusing to perform surgery based on the patient's hepatitis B status demonstrates a lack of understanding about the transmission routes and appropriate infection control measures in healthcare settings [24,25]. It is important to address this issue and educate healthcare providers about hepatitis B transmission, prevention, and appropriate precautions to ensure patient safety. Universal precautions, such as following standard protocols for infection control, the use of personal protective equipment, and proper sterilization techniques, can effectively minimize the risk of transmission of bloodborne pathogens, including hepatitis B, during surgical procedures [26-28]. This study's findings are consistent with those of earlier investigations into the attitudes and practices of healthcare providers about patient safety and quality of care. For instance, several studies indicated that the majority of healthcare personnel had favorable attitudes toward patient safety and quality of treatment, although there were some knowledge and practice gaps [29-31].

The findings from a current study regarding students’ attitudes and practices in various scenarios spark important discussions within the medical community. The results highlight the significance of reporting critical information, promoting transparency, and prioritizing patient safety [11,32]. The majority of participants demonstrated a commendable understanding of the need for interdisciplinary collaboration, acknowledging the input of colleagues in decision-making processes. The scenario involving a surgical complication emphasized the importance of accountability and taking responsibility for adverse events. Overall, these findings underscore the ongoing need for continuous education, professional development, and a patient-centered approach to enhance surgeon attitudes, promote ethical conduct, and foster a collaborative work environment. Through open discussions and proactive measures, healthcare organizations can strive toward a culture that prioritizes patient safety, effective teamwork, and professionalism among surgeons and healthcare professionals as a whole.

The present study possesses certain limitations that necessitate careful consideration when interpreting the results. The potential limitation of sample selection bias should be acknowledged, as the study exclusively targets medical students and interns affiliated with a particular university, which may restrict the applicability of the findings to a broader population. Additionally, the utilization of self-reported data obtained through surveys adds the possibility of recall bias and social desirability bias. Furthermore, the utilization of a cross-sectional design imposes limitations on the ability to establish causal links and observe temporal changes. The dissemination of the questionnaire via social media platforms has the potential to generate selection bias. It is imperative for future research endeavors to acknowledge these limitations in order to effectively address the existing gaps and enhance comprehension of the awareness and attitudes surrounding malpractice among medical students and interns.

Overall, this study's findings underline the significance of ensuring that medical students receive a thorough education on the distinctions between malpractice and surgical complications. This education should be an integral part of the medical curriculum and should be structured to ensure that students are not only able to distinguish between these two notions but also comprehend their legal and ethical ramifications.

## Conclusions

The findings of the current study showed that the clinical students and interns at Al-Baha University have a good level of knowledge and positive attitude toward patient safety, but there is a need to revise the curriculum to ensure that all students receive a uniform level of knowledge. Further research in this area may assist in uncovering the elements that contribute to inadequate knowledge and inform the creation of more effective educational interventions that can improve patient safety and healthcare delivery quality.
